# Lifestyle Habits and Alternative Tobacco and Nicotine Products: Results from the MINERVA Project

**DOI:** 10.3390/jcm15010389

**Published:** 2026-01-05

**Authors:** Giulia Lorenzoni, Honoria Ocagli, Danila Azzolina, Noor Muhammad Khan, Francesca Angioletti, Kostantina-Thaleia Pilali, Aslihan Şentürk Acar, Paola Berchialla, Matteo Martinato, Dario Gregori

**Affiliations:** 1 Unit of Biostatistics, Epidemiology and Public Health, Department of Cardiac, Thoracic, Vascular Sciences and Public Health, University of Padova, 35131 Padova, Italy; giulia.lorenzoni@unipd.it (G.L.); honoria.ocagli@unipd.it (H.O.); noor.khan@ubep.unipd.it (N.M.K.); francesca.angioletti@ubep.unipd.it (F.A.); konstantina.pilali@ubep.unipd.it (K.-T.P.); matteo.martinato@unipd.it (M.M.); 2 Department of Translational Medicine, University of Naples Federico II, 80131 Naples, Italy; danila.azzolina@unina.it; 3 Department of Actuarial Sciences, Hacettepe University, 06800 Ankara, Turkey; aslihansenturkacar@gmail.com; 4 Centre for Biostatistics, Epidemiology and Public Health, Department of Clinical and Biological Sciences, University of Torino, 10043 Orbassano, Italy; paola.berchialla@unito.it

**Keywords:** electronic nicotine delivery systems, heat-not-burn, dietary habits, lifestyle, cohort study

## Abstract

**Background/Objectives:** Alternative Tobacco and Nicotine Products (ATNPs) have gained widespread popularity. Although they are often promoted as lower-risk alternatives to traditional tobacco products, concerns remain regarding their association with risky behaviors among adolescents and young adults. This study examines the relationship between dietary and lifestyle habits and both ATNP use and intention to use ATNP among Italian participants in the MINERVA (My changINg lifEstyles our Research and eVeryone heAlth) international project. **Methods:** MINERVA is an observational, international, prospective cohort study. A study-specific questionnaire was administered to participants, who were recruited through informal snowball sampling. The questionnaire collected information on sociodemographic characteristics, lifestyle factors, dietary habits, and the use of both traditional tobacco products and ATNPs. Predictors of ATNP use and intention to use were assessed using logistic regression models. **Results:** Data from 7535 Italian participants were analyzed. Overall, 48% reported having ever used ATNP, and 14% of non-smokers and non-users expressed an intention to try these products. Significant predictors of ATNP use and intention to use included prior smoking, lower age, and having family members who smoke. Lifestyle factors such as frequent consumption of fast food, junk food, and alcoholic beverages were positively associated with both ATNP use and intention to use. Conversely, daily fruit and vegetable consumption was inversely associated with these outcomes. **Conclusions:** ATNP use and intention to use were associated with unhealthy dietary and lifestyle patterns. These findings highlight the importance of integrated public health strategies addressing substance use alongside broader lifestyle behaviors among adolescents and young adults.

## 1. Introduction

Alternative tobacco and nicotine products (ATNPs), i.e., electronic nicotine delivery systems (ENDS) like e-cigarettes and vape devices, heat-not-burn (HNB) tobacco, and oral nicotine, have gained great popularity in recent years [1,2]. They have been proposed as healthier alternatives to conventional tobacco products, because their use is associated with lower health risk compared to combustible products [3,4]. Additionally, they have the potential to serve as transition products to help with smoking cessation [5].

However, in a short time, these products have become very popular among adolescents and young adults [6] who do not necessarily smoke conventional tobacco products. It is noteworthy that, although their use has been associated with lower health risks than traditional products, they do not come without risks, especially for young people [7]. They are associated with nicotine dependence, to which adolescents seem to be particularly susceptible [8]. In addition, the use of these products in adolescents and young adults has been associated with traditional tobacco smoking progression and risky behaviors [9]. ATNP use is associated with alcohol consumption, heavy drinking, and marijuana use in adolescents and young adults [10].

Considering the potential for ATNP use to be associated with, or a predictor of, risky behaviors, there is an increasing interest in the literature in profiling ATNP users and people who intend to try ATNP.

It is not easy to summarize the literature in this field because of the methodological differences in the conduct of the published studies [11]. Nevertheless, all studies seem to agree on the predictors of ATNP use, regardless of the criteria used to select the sample and the instruments employed for assessment. Male gender, young age, and low socio-economic status (including low educational level) seem to be associated with a higher probability of using ATNP [12,13]. Among psychological factors, impulsivity traits and psychological distress have been suggested to be predictors of ATNP use [14].

Given the high prevalence of ATNP use in the adolescent age [6], several studies have concentrated on identifying predictors of use in this age group, showing that having already tried to smoke conventional products and exposure to family and friends who smoke are the strongest predictors of ATNP use [15,16]. Similar predictors have been identified regarding the intention to use ATNP [17].

Interestingly, the literature analysis highlights a knowledge gap. Recent studies have focused on socio-demographic characteristics and psychological factors. In contrast, only a few studies have attempted to characterize lifestyle habits according to ATNP use (or intention to use), with preliminary suggestions of an association between unhealthy eating and lifestyle habits [16,18]. Such a relationship must be better characterized, given the well-known strict mutual influence of dietary habits, lifestyle habits (including addictive behaviors), and socio-demographics. Literature suggests that eating patterns often coexist with other lifestyle behaviors. Young people who report irregular or unbalanced diets—including a higher consumption of protein-rich foods (PRFs) such as processed meats—also tend to show greater impulsivity, lower attention to overall diet quality, and a higher likelihood of engaging in risk behaviors, including smoking or vaping. These behaviors often cluster within the same family or peer environment, where dietary choices and experimentation with nicotine products may be shaped by similar social and behavioral influences [19].

In this context, the MINERVA (My changINg lifEstyles our Research and eVeryone heAlth) international project is ongoing to evaluate the association between dietary and lifestyle habits and the use of, and intention to use, ATNP. The present work, conducted within the context of the MINERVA study, focuses on Italian data to identify predictors of ATNP use and intention to use among non-smokers of traditional tobacco products.

## 2. Materials and Methods

The MINERVA is an observational, international, prospective cohort study. The project aims to evaluate the relationship between dietary and lifestyle factors with smoking habits, focusing specifically on ATNP use and intention to use.

To be eligible for inclusion, individuals must be aged 18–99 years, be able to read and complete the questionnaire, and accept the study’s privacy and participation policy. Individuals who experience difficulties in accessing, reading, or completing the questionnaire, or who are unable to provide the required consent, are not eligible for participation.

Study participants are enrolled employing an informal snowball sampling technique and administered with an online baseline questionnaire, following a standard methodology [20]. The questionnaire comprises various sections that assess socio-demographic characteristics, lifestyle habits, dietary behaviors, and smoking habits related to traditional smoking and ATNP use. The questionnaire is administered via LimeSurvey^©^, an open-source survey tool.

At the end of the questionnaire, subjects are asked to consent to being recontacted to be included in the cohort. If they consent to be involved in the cohort, they are asked to provide their email address and will be contacted twice a year to complete a follow-up questionnaire that records any changes in their smoking habits.

The study is international, involving Mediterranean countries (starting with Italy, then Turkey, and Greece), and it is ongoing. It follows the Declaration of Helsinki guidelines and was approved by the Bioethics Committee of the University of Turin (protocol 0075071) on 2 February 2024. All participants provide informed consent before participation, with the option to withdraw at any time. Consent to participate and to collect and process personal data is given electronically (by ticking the corresponding box) on the project website, after reading the detailed information on data collection and processing, and before accessing the questionnaire, in accordance with the European Commission General Data Protection Regulation (679/2016). Both the informed consent form for study participation and the data processing consent form have been approved by the institutional ethics committee.

The present work focuses on baseline Italian data, where follow-up is ongoing. The published study protocol details the subjects’ enrollment, questionnaire development, and distribution [21].

During the preparation of this manuscript, the authors used ChatGPT (OpenAI; GPT-4o) for the purposes of formal language revision, including grammar and syntax correction, improvement of clarity, and refinement of academic style.

### Statistical Analysis

Descriptive statistics were presented as median (I, III quartiles) for continuous variables and absolute numbers (percentages) for categorical variables.

Univariable logistic regression models were used to examine the relationship of socio-demographic characteristics, lifestyle habits, and dietary patterns with the ATNP use and intention to use. If the association was nonlinear, restricted cubic splines were used to estimate the models, and the change point was identified [22].

Complete case analysis was conducted, and statistical significance was defined as a *p*-value < 0.05. Analyses were performed using R [23] with the rms library.

## 3. Results

The MINERVA enrolled 7535 subjects in Italy (Table 1), and 4862 (65%) consented to be enrolled in the follow-up cohort. The median age was 42 years, with a slightly higher prevalence of females than males (53% vs. 47%). The analysis of socio-economic status revealed that 48% of the subjects held a high school diploma, while another 41% possessed a university degree. Three-fourths of the subjects (74%) were employed. The yearly income was generally medium-low, with 40% of the subjects reporting an annual income between 15,000 € and 30,000 €, and 30% reporting an annual income below 15,000 €.

Half of the subjects (46%) smoked traditional tobacco products daily, and 34% were past smokers. The median age of starting smoking among current and past smokers was 18 years (I–III quartiles: 15–20).

Regarding ATNP use, 48% (3645 subjects) have used an ATNP, with a median starting age of 32 years (I–III quartiles: 24–42 years). Of these, 2246 subjects reported being current users. The main reasons for using ATNP were to quit smoking tobacco (827 subjects) and the pleasure of using ATNP (855 subjects).

Among the 5289 subjects who were not current ATNP users, 25% stated that they were willing to try an ATNP product.

### 3.1. Predictors of ATNP Use Intention

The analysis involved 3214 subjects who did not smoke traditional tobacco products daily and did not currently use ATNP. Among them, 447 (14%) indicated they were open to using ATNP (Table 2).

**Figure 1:**
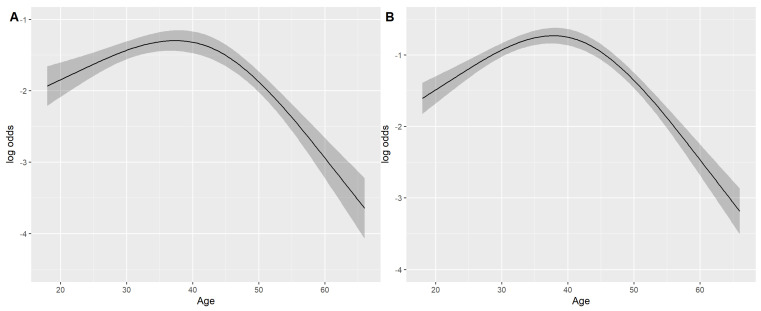
Relationship between age and Alternative Tobacco and Nicotine Products intention to use (**A**) and Alternative Tobacco and Nicotine Products use (**B**). The association was estimated using univariable logistic regression model with restricted cubic splines.

Among socio-demographic characteristics, age was significantly associated with the intention of trying. Interestingly, this association was nonlinear (*p*-value < 0.001), with a change point at 45 years of age (Figure 1A). Specifically, the likelihood of trying ATNP increased with age up to age 45.

As concerns smoking history, past smokers were significantly more likely to be interested in trying ATNP (OR 1.48, 95% CI 1.09–1.99), and the older they had started to smoke, the more likely they were to use ATNP (OR 1.04, 95% CI 1.01–1.06). Not least, the smoking family environment was found to be a relevant predictor of interest in ATNP, with subjects having family members who smoke found to have a higher likelihood of trying ATNP (OR 4.39, 95% CI 3.55–5.44).

Lifestyle and dietary habits were significant predictors of the intention to try ATNPs. Being unaware of the impact of the diet on physical health and not paying attention to the diet were significant predictors of the intention to use ATNP (ORs: 3.79, 95% CI 1.50–8.97, and 2.13, 95% CI 1.11–3.87, respectively). Drinking alcoholic beverages and eating junk food, e.g., French fries, cookies, and candies, as snacks were associated with a higher likelihood of trying ATNP (ORs: 1.77, 95% CI 1.32–2.42, and 1.61, 95% CI 1.29–2.02, respectively). Similarly, eating fast food at least once a week was associated with interest in ATNP (OR 2.67, 95% CI 2.01–3.52). Conversely, daily vegetable intake was protective against the intention to use ATNP (OR 0.75, 95% CI 0.60–0.93).

### 3.2. Predictors of ATNP Use

The analysis involved 4104 subjects who did not smoke traditional tobacco products daily (Table 3). Among them, 890 (22%) were ATNP users.

The predictors of ATNP use were similar to ATNP intention to use. A significant, non-linear relationship (*p*-value < 0.001) with age was found (Figure 1B), with a change point identified at 42 years. Interestingly, even though ATNP users were significantly younger than non-ATNP users, i.e., a median of 38 (I–III quartiles: 29–46) vs. 43 (I–III quartiles: 29–56) years of age, respectively, they were significantly more likely to be past smokers (84% vs. 26%).

Among dietary habits, consuming junk food snacks (OR 1.51, 95% CI 1.28, 1.79) and alcohol (OR 3.18, 95% CI 2.42, 4.27), and eating food from fast food restaurants at least once a week (2.22, 95% CI 1.80, 2.73) were significantly associated with a higher likelihood of ATNP use. Conversely, people who ate fruit regularly were less likely to use ATNPs.

## 4. Discussion

In light of the recent spread of ATNPs among teenagers and young adults, characterizing ATNP use and intention to use would be helpful from a public health perspective. It is noteworthy that ATNPs come with fewer health risks compared to traditional smoking. However, it has been suggested that they could act as an intermediate step to the use of conventional tobacco products in non-smokers. Studies have shown that adolescents and young adults who use e-cigarettes or other ATNPs are more likely to subsequently start smoking traditional cigarettes than non-users [24], even after adjusting for baseline susceptibility and psychosocial determinants. This transition is often interpreted within the gateway hypothesis, whereby early exposure to nicotine, normalization of smoking-related behaviors, and reinforcement through peer contexts may facilitate the transition to combustible tobacco use. In parallel, ATNP use has been repeatedly linked to other risk behaviors [25,26], suggesting that vaping and similar products may be embedded in broader behavioral trajectories. These findings provide a conceptual basis for interpreting ATNP use not as an isolated behavior but as part of a broader set of risk patterns emerging among young adults.

The results of the present study showed that predictors of ATNP use and intention to use are similar to each other and include young age, unhealthy eating habits, and a positive family or personal smoking history. Evidence from Italian and European studies aligns with the present findings, showing that ATNP use is common in younger populations: Italian and European surveys have reported high prevalence of vaping among adolescents and young adults [27,28].

It would not be easy to compare results from different studies. Each published research study has analyzed different populations, e.g., including only non-smokers, or both smokers and non-smokers. Furthermore, each study defines the outcome and the products under study differently. Some studies focused solely on ATNP use, while others examined the willingness to try ATNPs. Furthermore, most studies evaluated heated tobacco products, while others assessed e-cigarettes. Despite these differences, some common predictors of ATNP use or intention to use could be identified, such as young age, male gender, and being a smoker (or having family members who smoke) [15,16]. These findings are consistent with those of the present study, showing a strong, nonlinear, significant relationship with age. In contrast, no significant associations with gender were detected. Together with socio-demographic characteristics, past smoking history was also a significant predictor of both ATNP use and intention to use, with a markedly more pronounced effect for ATNP use.

Regarding the relationship between ATNP use and dietary domains, the literature provides limited evidence. A few recent studies have suggested that vaping seems to be used as a weight control measure among young people [29] and seems to be associated with eating disorders, including binge eating, anorexia nervosa, and bulimia [30,31], even though the evidence is controversial [32]. The main novelty of the present study was the analysis of diet quality in relation to ATNPs. Interestingly, junk food, alcohol, and fast-food consumption were all associated with a higher likelihood of trying and using ATNPs. These findings seem to reinforce the hypothesis that the use of ATNPs is related to unhealthy behaviors, including eating behaviors, as suggested by previous studies [16].

The associations observed between ATNP use, intention to use them, and unhealthy eating habits and lifestyles are plausible from a behavioral perspective. First, these behaviors may share common psychological determinants, such as impulsivity, sensation seeking, or reduced self-regulation, which may predispose individuals to both risky eating patterns and experimentation with nicotine products. Second, family and peer environments that normalize smoking or ATNP use may also promote less structured or less healthy eating habits, reinforcing the clustering of these behaviors. Furthermore, alcohol and fast food consumption are often associated with social occasions where tobacco and ATNP use are more likely, providing additional opportunities for co-occurrence. Although a direct biological link between specific dietary components and ATNP use cannot be established in this study, the observed pattern is consistent with a broader risk-behavior profile in which dietary choices, substance use, and lifestyle habits are interconnected.

### 4.1. Public Health Implications

These findings suggest that ATNP prevention should not be considered separately from other lifestyle behaviors. School-based prevention programs that address smoking, ATNP use, and nutrition jointly, rather than in separate educational tracks, may be more effective in reaching youth who tend to accumulate multiple risk behaviors. Integrating nutrition education with tobacco and nicotine prevention through coordinated programs, counseling, and peer-led initiatives could help identify and support individuals with overall “unhealthy” lifestyles. Furthermore, the clustering of ATNP use with other unhealthy behaviors reinforces the importance of comprehensive tobacco control policies, including strict regulation of marketing and flavors, enforcement of age limits, and sustained public information campaigns that explicitly address ATNPs alongside conventional tobacco products. Such integrated strategies may be particularly relevant in settings where ATNPs are perceived as less harmful and are widely accessible to young adults.

### 4.2. Study Limitations

The study’s main limitation is the cross-sectional nature of the current data analysis, which is also a limitation of most research investigating potential predictors of ATNP use. The topic is relatively new, and cohort studies require time to develop. Furthermore, the use of a self-administered questionnaire may introduce recall and social desirability biases, particularly for sensitive behaviors such as smoking and eating habits. Although large, the study population is not representative, as recruitment was conducted using an informal snowball sampling method. Finally, the questionnaire did not include a qualitative assessment of motivations or intentions to use ATNPs, limiting the ability to explore the contextual or psychosocial factors underlying these behaviors.

## 5. Conclusions

This study provides evidence about a general “unhealthy” environment characterizing ATNP use or willingness to use. Therefore, a clear separation of health determinants among ATNP use, diet, physical activity, and other demographic and lifestyle aspects is complex. In this context, the cross-sectional design limits the ability to draw causal conclusions. Future longitudinal follow-up within the MINERVA cohort will provide a better understanding of how these factors evolve in relation to ATNP use.

For public health implications, these findings highlight the need for prevention strategies that address nutrition, lifestyle behaviors, and tobacco and nicotine use in an integrated manner, rather than treating these domains in isolation. Combining nutritional education with tobacco prevention initiatives may be particularly effective for reaching individuals who tend to engage in multiple risk-prone behaviors.

## Figures and Tables

**Table 1:** Sample characteristics. Data are absolute numbers (percentages) and median (I, III quartiles).

Characteristic	N	N = 7535
*Socio-demographic characteristics*		
Gender	7524	
Female		3953 (53%)
Male		3571 (47%)
Age	7535	42 (32, 52)
Education	7535	
High school diploma		3604 (48%)
Primary/Middle school		843 (11%)
University degree		3088 (41%)
Job	7535	
Unemployed		1404 (19%)
Student		540 (7.2%)
Employed		5591 (74%)
Yearly gross income	7480	
>50,000		621 (8.3%)
0–15,000		2213 (30%)
15,000–30,000		2970 (40%)
30,000–50,000		1676 (22%)
Number of adult family members	7535	2.00 (2.00, 3.00)
Children in the family	7535	
No		5501 (73%)
Yes		2034 (27%)
Teenagers in the family	7535	
No		5915 (79%)
Yes		1620 (21%)
*Smoking and ATNP use habits*		
Smoker	7535	
No		4104 (54%)
Yes		3431 (46%)
Smokers in the family	7535	
No		2225 (30%)
Yes		5310 (70%)
Past smoker	2992	
No		1982 (66%)
Yes		1010 (34%)
Age started smoking	5553	18.0 (15.0, 20.0)
Ever used ATNP	7535	
No		3890 (52%)
Yes		3645 (48%)
Current ATNP user	3645	
No		1399 (38%)
Yes		2246 (62%)
Age started using ATNP	3645	32 (24, 42)
Reasons for ATNP use (actual users only) *	3902	
To quit smoking tobacco.		827 (100%)
To avoid returning to smoking tobacco		473 (100%)
Enjoy it		855 (100%)
Addicted to it		168 (100%)
Use it in situations where smoking tobacco is not allowed		507 (100%)
Believe it is less harmful than smoking tobacco		637 (100%)
Prefer the available flavors		356 (100%)
Influenced by a friend or family member		79 (100%)
Intention to try ATNP (only for non-users)	5289	
No		3967 (75%)
Yes		1322 (25%)
*Eating habits*		
Junk food snacks	7535	
No		2283 (30%)
Yes		5252 (70%)
Alcoholic beverages consumption	7535	
No		900 (12%)
Yes		6635 (88%)
Daily fruit consumption	7535	
No		2471 (33%)
Yes		5064 (67%)
Daily vegetables consumption	7535	
No		2104 (28%)
Yes		5431 (72%)
Weekly fast food visit	7535	
No		6483 (86%)
Yes		1052 (14%)
Number of daily meals	7535	
1		432 (5.7%)
2		747 (9.9%)
3		3155 (42%)
4		1815 (24%)
5		1386 (18%)
Eating habits description	7535	
I try to be careful about what I eat		2637 (35%)
I try to be careful, but I don’t always succeed		3934 (52%)
I don’t particularly worry about what I eat		793 (11%)
I eat everything without thinking		171 (2.3%)
Impact of eating habits on physical shape	7535	
Very much		3366 (45%)
Enough		3822 (51%)
Little		298 (4.0%)
Not at all		49 (0.7%)
*Lifestyle habits*		
Wake-up time	7535	
At 6.30 or earlier		2495 (33%)
Around 7.00		2062 (27%)
Around 7.30		1449 (19%)
After 8.00		1032 (14%)
I don’t have a specific time		497 (6.6%)
Bedtime	7535	
At 10.30 pm or earlier		1554 (21%)
Around 11.00 pm		3197 (42%)
After midnight		2196 (29%)
I don’t have a specific time		588 (7.8%)
Effect of sleep on physical health	7535	
Very much		3760 (50%)
Enough		3471 (46%)
Little		268 (3.6%)
Not at all		36 (0.5%)
Attention to physical shape	7535	
Very much		1349 (18%)
Enough		4579 (61%)
Little		1488 (20%)
Not at all		119 (1.6%)
Regular physical activity	7535	
No		3068 (41%)
Yes		4467 (59%)

ATNP: Alternative Tobacco and Nicotine Product. * The percentages do not sum to 100% because respondents could select more than one reason.

**Table 2:** Characteristics of the subjects who did not smoke traditional tobacco products daily and did not currently use Alternative Tobacco and Nicotine Products according to Alternative Tobacco and Nicotine Products intention to use and results of the univariable logistic regression analysis. Data are absolute numbers (percentages) and median (I, III quartiles). Results of the regression analysis are reported as OR, 95% CI, and *p*-value.

Characteristic	N	No Intention to Use ATNP N = 2767	Intention to Use ATNP N = 447	OR	95% CI	*p*-Value
*Socio-demographic characteristics*						
Gender	3208					
Female		1511 (55%)	235 (53%)			
Male		1252 (45%)	210 (47%)	1.08	0.88, 1.32	0.5
Age	3214	44 (30, 57)	37 (28, 47)	0.97	0.97, 0.98	<0.001
Education	3214					
High school diploma		1315 (48%)	198 (44%)			
Primary/Middle school		309 (11%)	43 (9.6%)	0.92	0.64, 1.30	0.7
University degree		1143 (41%)	206 (46%)	1.2	0.97, 1.48	0.094
Job	3214					
Unemployed		641 (23%)	66 (15%)			
Student		308 (11%)	43 (9.6%)	1.36	0.90, 2.03	0.14
Employed		1818 (66%)	338 (76%)	1.81	1.38, 2.40	<0.001
Yearly gross income	3180					
>50,000		204 (7.5%)	27 (6.1%)			
0–15,000		872 (32%)	142 (32%)	1.23	0.81, 1.94	0.4
15,000–30,000		1060 (39%)	164 (37%)	1.17	0.77, 1.84	0.5
30,000–50,000		600 (22%)	111 (25%)	1.4	0.90, 2.23	0.14
Number of adult family members	3214	2.00 (2.00, 3.00)	2.00 (2.00, 3.00)	1.01	0.95, 1.07	0.6
Children in family	3214					
No		2265 (82%)	284 (64%)			
Yes		502 (18%)	163 (36%)	2.59	2.08, 3.21	<0.001
Teenagers in family	3214					
No		2312 (84%)	334 (75%)			
Yes		455 (16%)	113 (25%)	1.72	1.35, 2.17	<0.001
*Smoking and ATNP use habits*						
Smokers in family	3214					
No		1876 (68%)	145 (32%)			
Yes		891 (32%)	302 (68%)	4.39	3.55, 5.44	<0.001
Past smoker	2576					
No		1773 (75%)	142 (67%)			
Yes		591 (25%)	70 (33%)	1.48	1.09, 1.99	0.011
Age started smoking	1299	17.0 (15.0, 20.0)	18.0 (16.0, 20.0)	1.04	1.01, 1.06	0.002
Ever used ATNP	3214					
No		2401 (87%)	296 (66%)			
Yes		366 (13%)	151 (34%)	0.3	0.24, 0.37	<0.001
Age started using ATNP	517	26 (20, 39)	25 (20, 35)	0.99	0.97, 1.01	0.3
*Eating habits*						
Junk food snacks	3214					
No		1012 (37%)	118 (26%)			
Yes		1755 (63%)	329 (74%)	1.61	1.29, 2.02	<0.001
Alcoholic beverages consumption	3214					
No		523 (19%)	52 (12%)			
Yes		2244 (81%)	395 (88%)	1.77	1.32, 2.42	<0.001
Daily fruit consumption	3214					
No		769 (28%)	135 (30%)			
Yes		1998 (72%)	312 (70%)	0.89	0.72, 1.11	0.3
Daily vegetables consumption	3214					
No		705 (25%)	140 (31%)			
Yes		2062 (75%)	307 (69%)	0.75	0.60, 0.93	0.009
Weekly fast food visit	3214					
No		2558 (92%)	367 (82%)			
Yes		209 (7.6%)	80 (18%)	2.67	2.01, 3.52	<0.001
Number of daily meals		3.00 (3.00, 4.00)	3.00 (3.00, 4.00)	0.85	0.77, 0.93	<0.001
Eating habits description	3214					
I try to be careful about what I eat		1053 (38%)	147 (33%)			
I try to be careful, but I don’t always succeed		1410 (51%)	241 (54%)	1.22	0.98, 1.53	0.071
I don’t particularly worry about what I eat		257 (9.3%)	45 (10%)	1.25	0.87, 1.79	0.2
I eat everything without thinking		47 (1.7%)	14 (3.1%)	2.13	1.11, 3.87	0.017
Impact of eating habits on physical shape	3214					
Very much		1233 (45%)	186 (42%)			
Enough		1395 (50%)	238 (53%)	1.13	0.92, 1.39	0.2
Little		125 (4.5%)	15 (3.4%)	0.8	0.44, 1.35	0.4
Not at all		14 (0.5%)	8 (1.8%)	3.79	1.50, 8.97	0.003
*Lifestyle habits*						
Wake-up time	3214					
At 6.30 or earlier		886 (32%)	121 (27%)			
Around 7.00		768 (28%)	127 (28%)	1.21	0.93, 1.58	0.2
Around 7.30		543 (20%)	97 (22%)	1.31	0.98, 1.74	0.067
After 8.00		393 (14%)	67 (15%)	1.25	0.90, 1.72	0.2
I don’t have a specific time		177 (6.4%)	35 (7.8%)	1.45	0.95, 2.16	0.076
Bedtime	3214					
At 10.30 pm or earlier		633 (23%)	114 (26%)			
Around 11.00 pm		1209 (44%)	179 (40%)	0.82	0.64, 1.06	0.13
After midnight		695 (25%)	114 (26%)	0.91	0.69, 1.21	0.5
I don’t have a specific time		230 (8.3%)	40 (8.9%)	0.97	0.65, 1.42	0.9
Effect of sleep on physical health	3214					
Very much		1371 (50%)	213 (48%)			
Enough		1270 (46%)	217 (49%)	1.1	0.90, 1.35	0.4
Little		114 (4.1%)	11 (2.5%)	0.62	0.31, 1.12	0.14
Not at all		12 (0.4%)	6 (1.3%)	3.22	1.11, 8.38	0.021
Attention to physical shape	3214					
Very much		424 (15%)	104 (23%)			
Enough		1731 (63%)	273 (61%)	0.64	0.50, 0.83	<0.001
Little		569 (21%)	63 (14%)	0.45	0.32, 0.63	<0.001
Not at all		43 (1.6%)	7 (1.6%)	0.66	0.27, 1.43	0.3
Regular physical activity	3214					
No		1190 (43%)	161 (36%)			
Yes		1577 (57%)	286 (64%)	1.34	1.09, 1.65	0.006

ATNP: Alternative Tobacco and Nicotine Product.

**Table 3:** Characteristics of the subjects who did not smoke traditional tobacco products daily according to Alternative Tobacco and Nicotine Products use and results of the univariable logistic regression analysis. Data are absolute numbers (percentages) and median (I–III quartiles). Results of the regression analysis are reported as OR, 95% CI, and *p*-value.

Characteristic	N	No ATNP Users N = 3214	ATNP Users N = 890	OR	95% CI	*p*-Value
*Socio-demographic characteristics*						
Gender	4097					
Female		1746 (54%)	501 (56%)			
Male		1462 (46%)	388 (44%)	0.92	0.80, 1.07	0.3
Age	4104	43 (29, 56)	38 (29, 46)	0.98	0.97, 0.98	<0.001
Education	4104					
High school diploma		1513 (47%)	403 (45%)			
Primary/Middle school		352 (11%)	92 (10%)	0.98	0.76, 1.26	0.9
University degree		1349 (42%)	395 (44%)	1.1	0.94, 1.29	0.2
Job	4104					
Unemployed		707 (22%)	127 (14%)			
Student		351 (11%)	72 (8.1%)	1.14	0.83, 1.56	0.4
Employed		2156 (67%)	691 (78%)	1.78	1.45, 2.20	<0.001
Yearly gross income	4062					
>50,000		231 (7.3%)	55 (6.2%)			
0–15,000		1014 (32%)	280 (32%)	1.16	0.85, 1.61	0.4
15,000–30,000		1224 (38%)	374 (42%)	1.28	0.94, 1.77	0.12
30,000–50,000		711 (22%)	173 (20%)	1.02	0.73, 1.44	0.9
Number of adult family members	4104	2.00 (2.00, 3.00)	2.00 (2.00, 3.00)	0.98	0.93, 1.02	0.3
Children in family	4104					
No		2549 (79%)	588 (66%)			
Yes		665 (21%)	302 (34%)	1.97	1.67, 2.32	<0.001
Teenagers in family	4104					
No		2646 (82%)	700 (79%)			
Yes		568 (18%)	190 (21%)	1.26	1.05, 1.52	0.013
*Smoking and ATNP use habits*						
Smokers in family	4104					
No		2021 (63%)	100 (11%)			
Yes		1193 (37%)	790 (89%)	13.4	10.8, 16.8	<0.001
Past smoker	2992					
No		1915 (74%)	67 (16%)			
Yes		661 (26%)	349 (84%)	15.1	11.5, 20.0	<0.001
Age started smoking	2122	18.0 (16.0, 20.0)	17.0 (15.0, 20.0)	0.98	0.96, 1.00	0.025
*Eating habits*						
Junk food snacks	4104					
No		1130 (35%)	235 (26%)			
Yes		2084 (65%)	655 (74%)	1.51	1.28, 1.79	<0.001
Alcoholic beverages consumption	4104					
No		575 (18%)	57 (6.4%)			
Yes		2639 (82%)	833 (94%)	3.18	2.42, 4.27	<0.001
Daily fruit consumption	4104					
No		904 (28%)	314 (35%)			
Yes		2310 (72%)	576 (65%)	0.72	0.61, 0.84	<0.001
Daily vegetable consumption	4104					
No		845 (26%)	230 (26%)			
Yes		2369 (74%)	660 (74%)	1.02	0.87, 1.21	0.8
Weekly fast-food visit	4104					
No		2925 (91%)	730 (82%)			
Yes		289 (9.0%)	160 (18%)	2.22	1.80, 2.73	<0.001
Number of daily meals	4104	3.00 (3.00, 4.00)	3.00 (3.00, 4.00)	0.9	0.84, 0.96	0.001
Eating habits description	4104					
I try to be careful about what I eat		1200 (37%)	301 (34%)			
I try to be careful, but I don’t always succeed		1651 (51%)	482 (54%)	1.16	0.99, 1.37	0.066
I don’t particularly worry about what I eat		302 (9.4%)	93 (10%)	1.23	0.94, 1.59	0.13
I eat everything without thinking		61 (1.9%)	14 (1.6%)	0.91	0.49, 1.61	0.8
Impact of eating habits on physical shape	4104					
Very much		1419 (44%)	391 (44%)			
Enough		1633 (51%)	448 (50%)	1	0.85, 1.16	>0.9
Little		140 (4.4%)	45 (5.1%)	1.17	0.81, 1.65	0.4
Not at all		22 (0.7%)	6 (0.7%)	0.99	0.36, 2.31	>0.9
*Lifestyle habits*						
Wake-up time	4104					
At 6.30 or earlier		1007 (31%)	248 (28%)			
Around 7.00		895 (28%)	243 (27%)	1.1	0.90, 1.34	0.3
Around 7.30		640 (20%)	186 (21%)	1.18	0.95, 1.46	0.13
After 8.00		460 (14%)	149 (17%)	1.32	1.04, 1.66	0.02
I don’t have a specific time		212 (6.6%)	64 (7.2%)	1.23	0.89, 1.67	0.2
Bedtime	4104					
At 10.30 pm or earlier		747 (23%)	162 (18%)			
Around 11.00 pm		1388 (43%)	381 (43%)	1.27	1.03, 1.56	0.024
After midnight		809 (25%)	277 (31%)	1.58	1.27, 1.97	<0.001
I don’t have a specific time		270 (8.4%)	70 (7.9%)	1.2	0.87, 1.63	0.3
Effect of sleep on physical health	4104					
Very much		1584 (49%)	441 (50%)			
Enough		1487 (46%)	422 (47%)	1.02	0.88, 1.19	0.8
Little		125 (3.9%)	26 (2.9%)	0.75	0.47, 1.14	0.2
Not at all		18 (0.6%)	1 (0.1%)	0.2	0.01, 0.97	0.12
Attention to physical shape	4104					
Very much		528 (16%)	179 (20%)			
Enough		2004 (62%)	543 (61%)	0.8	0.66, 0.97	0.024
Little		632 (20%)	157 (18%)	0.73	0.57, 0.93	0.012
Not at all		50 (1.6%)	11 (1.2%)	0.65	0.31, 1.23	0.2
Regular physical activity	4104					
No		1351 (42%)	332 (37%)			
Yes		1863 (58%)	558 (63%)	1.22	1.05, 1.42	0.011

ATNP: Alternative Tobacco and Nicotine Product.

## Data Availability

The datasets presented in this article are not readily available because the data are part of an ongoing study.
